# Combination chemotherapy versus temozolomide for patients with methylated MGMT (m-MGMT) glioblastoma: results of computational biological modeling to predict the magnitude of treatment benefit

**DOI:** 10.1007/s11060-021-03780-0

**Published:** 2021-06-08

**Authors:** Michael Castro, Anusha Pampana, Aftab Alam, Rajan Parashar, Swaminathan Rajagopalan, Deepak Anil Lala, Kunal Ghosh Ghosh Roy, Sayani Basu, Annapoorna Prakash, Prashant Nair, Vishwas Joseph, Ashish Agarwal, Poornachandra G, Liptimayee Behura, Shruthi Kulkarni, Nikita Ray Choudhary, Shweta Kapoor

**Affiliations:** 1 Personalized Cancer Medicine PLLC, 1735 S Hayworth Ave., Los Angeles, CA USA; 2 Cellworks Group, Inc., S. San Francisco, CA USA; 3 Cellworks Group, Inc., Bangalore, India

**Keywords:** Computational biological modeling, Biosimulation, Precision medicine, Glioblastoma (GBM), O^6^-methylguanine-DNA methyl-transferase **(**MGMT), Artificial intelligence (AI)

## Abstract

**Background:**

A randomized trial in glioblastoma patients with *methylated-MGMT* (*m-MGMT*) found an improvement in median survival of 16.7 months for combination therapy with temozolomide (TMZ) and lomustine, however the approach remains controversial and relatively under-utilized. Therefore, we sought to determine whether comprehensive genomic analysis can predict which patients would derive large, intermediate, or negligible benefits from the combination compared to single agent chemotherapy.

**Methods:**

Comprehensive genomic information from 274 newly diagnosed patients with methylated*-MGMT* glioblastoma (GBM) was downloaded from TCGA. Mutation and copy number changes were input into a computational biologic model to create an avatar of disease behavior and the malignant phenotypes representing hallmark behavior of cancers. In silico responses to TMZ, lomustine, and combination treatment were biosimulated. Efficacy scores representing the effect of treatment for each treatment strategy were generated and compared to each other to ascertain the differential benefit in drug response.

**Results:**

Differential benefits for each drug were identified, including strong, modest-intermediate, negligible, and deleterious (harmful) effects for subgroups of patients. Similarly, the benefits of combination therapy ranged from synergy, little or negligible benefit, and deleterious effects compared to single agent approaches.

**Conclusions:**

The benefit of combination chemotherapy is predicted to vary widely in the population. Biosimulation appears to be a useful tool to address the disease heterogeneity, drug response, and the relevance of particular clinical trials observations to individual patients. Biosimulation has potential to spare some patients the experience of over-treatment while identifying patients uniquely situated to benefit from combination treatment. Validation of this new artificial intelligence tool is needed.

**Supplementary Information:**

The online version contains supplementary material available at 10.1007/s11060-021-03780-0.

## Background

In the landmark trial by Stupp and colleagues in glioblastoma (GBM), adjuvant temozolomide (TMZ) chemotherapy decreased the death rate compared to radiation therapy alone by 37% and in 2005 became the standard of care for newly diagnosed patients [1]. By alkylating guanine residues, TMZ induces futile DNA mismatch repair that introduces single-stranded breaks resulting in replication fork arrest and cell death. The dominant but by no means only mechanism of TMZ resistance is caused by the DNA repair enzyme O^6^-methylguanine-DNA methyl-transferase **(**MGMT) which removes alkylated guanine bases to counter the lethal effects of the drug. The *MGMT* gene is subject to epigenetic regulation via methylation which silences its transcription. Hence, cancers with *MGMT* methylation (*m-MGMT*) tend to be highly responsive to TMZ with an increase in median survival of approximately six and one-half months compared to less than one month for unmethylated-*MGMT* (*u-MGMT*) cancers [2].

For patients with *m-MGMT*, efforts to build on the benefits of TMZ have explored combination chemotherapy regimens with the addition of lomustine to the TMZ backbone. A single arm, phase II trial (UKT-03) evaluated TMZ plus lomustine in newly diagnosed GBM patients revealed a median survival of 23 months, considerably better than the historical experience of 14.6 months [3]. Larger benefits accrued in the *m-MGMT* patients with a 2-year survival of 75% compared to 20% for *u-MGMT* patients, and median survival of not reached and 12.6 months, respectively. This led to the Nordic phase III trial (NOA-9) in newly diagnosed, *m-MGMT* GBM which randomized 141 patients to standard therapy (RT-TMZ followed by adjuvant TMZ) or experimental therapy with radiation alone followed by lomustine and TMZ every 6 weeks [4]. A striking superiority for the combination was observed for overall survival: 48.1 vs. 31.4 months for single agent TMZ (hazard ratio [HR] 0·60, 95% CI 0·35–1·03, p = 0·0492). A significant overall survival difference was also found in a secondary analysis of the intention-to-treat population (n = 141, HR 0·60, 95% CI 0·35–1·03, p = 0·0432). However, neurooncologists are often unwilling to prescribe combination therapy citing the small study size, delayed separation of the progression free survival curves, and increased toxicity in the combination arm [5].

Nevertheless, combination therapy may represent a survival opportunity for some patients and raises the question whether deeper genomic interrogation can identify the magnitude of benefit for combination treatment compared to single agent chemotherapy. Besides MGMT, differential sensitivity to both TMZ and lomustine is based on combinations of DNA repair abnormalities, impaired DNA checkpoints, epigenetic dysregulation, and oncogenic signaling pathways has been described [6–21]. These insights suggest we should be able to do a better job of stratifying patients by incorporating a more complete molecular diagnosis into therapeutic decision making. At the same time, the complexity of integrating the consequences of dozens of genomic abnormalities governing growth, apoptosis, and DNA repair is daunting. Accordingly, *biosimulation* based on comprehensive signaling pathway impact analysis utilizes computational biology modeling (CBM) of nearly 4,000 proteins. A virtual *avatar* of the patient’s cancer can be generated from comprehensive genomic inputs that permits an interrogation of the disease network regarding the impact size of various drug combinations on the hallmark behaviors of cancer. CBM incorporates DNA repair pathways (i.e., BER, MMR, HRR, NHEJ), apoptosis, survival, proliferation, oxidative stress, DNA checkpoints, receptor tyrosine kinases, transcription factors, and the TP53, NFKB, and hedgehog pathways. CBM has demonstrated high positive and negative predictive value (~ 90%) for predicting clinical outcomes in patients with low grade glioma, [22] glioblastoma [23, 24] and recently has received increasing attention for cancers that pose challenges to the development of new treatment approaches [21, 22, 25]. In a population of 100 patients with glioblastoma, biosimulation of treatment response was found to be strongly predictive of disease free survival (p = 0.0266) and overall survival (p = 0.0125), offering evidence that validates biosimulation [26]. Accordingly, we sought to assess the anticipated outcomes of *m-MGMT* GBM patients treated with either TMZ, lomustine, or the combination. As such the study was undertaken to assess the landscape of predicted responses based on comprehensive molecular diagnosis, but does not set out to validate the predictions with individual survival outcomes.

## Materials and methods

### Patient genomic information

The myCare -015 cohort consisted of adult patients more than 18 years of age with newly diagnosed *m-MGMT* GBM. Mutation and copy number information was downloaded from TCGA (N = 274) from https://www.cbioportal.org/.

### Details of CBM and in silico modeling approach

Biosimulation derives the signaling pathway impact from the totality of genomic aberrations represented by mutations and copy number aberrations to determine the consequences for hallmark behaviors of cancer, including proliferation, apoptosis, oxidative stress, immune evasion, etc. and a composite malignant phenotype represented by cell number (See Supplementary Sect. 1 for additional details).

For each patient, treatment efficacy scores (T_eff_) were computed for TMZ, lomustine, and the combination of both drugs based on their ability to reverse the *phenotypic changes* driving disease progression (i.e. proliferation) and to produce cell death (i.e. DNA damage, reactive oxygen species, survival, apoptotic blockade). In essence, computational modeling of genomic aberrations and the dysregulated signaling pathways that follow permits a *quantitative determination* of various phenotypic behaviors of each individual’s cancer. The magnitude of divergence of each phenotype from the healthy cell characterizes the disease state and drug response for each patient which varies across the population according the genomic abnormalities represented in the model. For example, some patients’ cancers are characterized by homologous recombination repair deficiency or DNA checkpoint defects which enhance responsiveness to chemotherapy. Others have mismatch repair deficits that compromises the benefit of temozolomide. Some patients have epigenetic aberrations that result in a highly proliferative cancer, while others may have signaling pathway dysregulation leading to a greater defect in apoptosis or enhancement of survival. Still others have upregulated multidrug resistance pumps that eliminate chemotherapy from the cell. In most cases, patients have multiple abnormalities which alter drug response in complex ways.

Once the disease avatar has been created, the interaction of temozolomide, lomustine, and the combination of both drugs with the unique disease network of each cancer is biosimulated to determine the quantitative impact of treatment on the various disease phenotypes. CBM integrates the sum of these interactions across the each patient’s disease network to produce a measurement of the extent to which the disease drivers are defeated by the treatment. In this way, CBM measures the degree of drug resistance and sensitivity in each patient, the basis of which varies considerably in the population.

The *composite phenotype*, T_eff_, denoting cell number represents the product of the individual phenotypic behaviors. As such, T_eff_ represents the simulated impact on tumor growth caused by each therapy under consideration. Low T_eff_ (< 30) represents lack of treatment efficacy. Higher T_eff_ scores represent a collapse of phenotypic behaviors that represent the disease state. T_eff_ > 80 indicates and exceptional responder. T_eff_ < 0 suggests the treatment accelerates the disease state.

## Results

### Patient characteristics

**Fig. 1:**
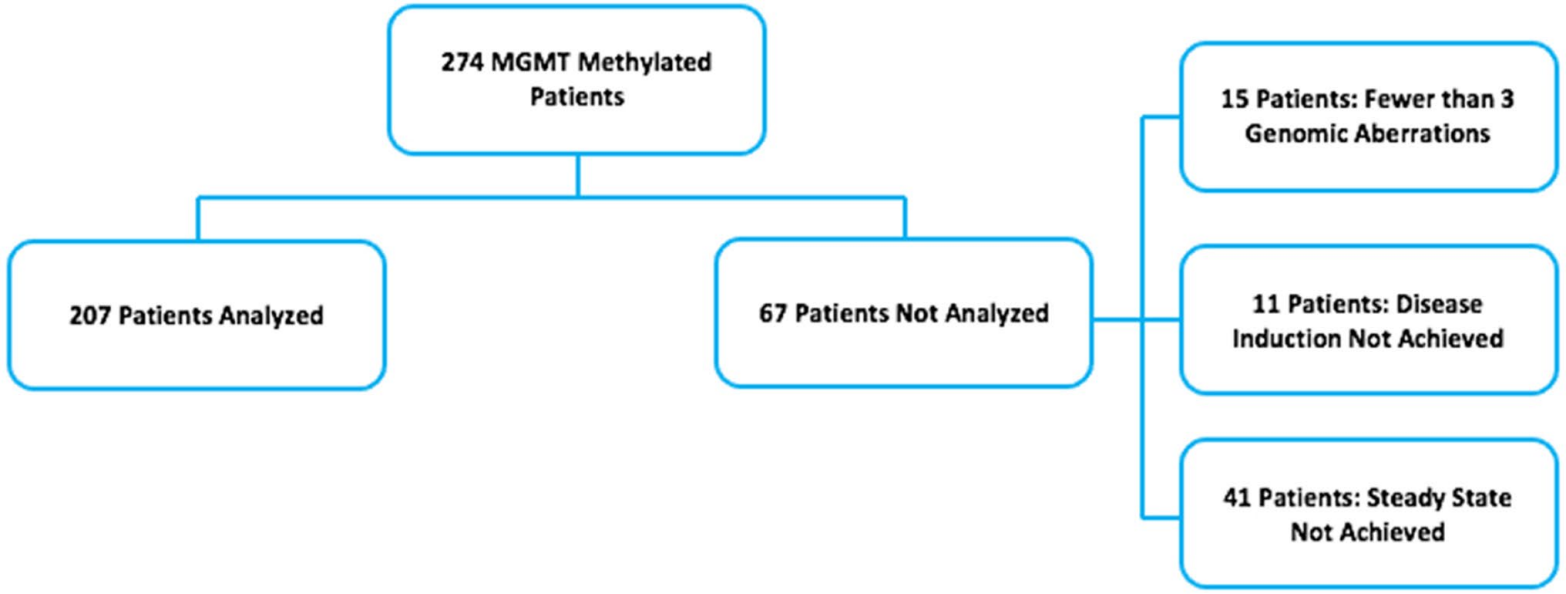
TCGA patients available for analysis. A minimum requirement of three genomic aberrations is needed to enable actionable insights and predictions (15/67). In most cases it was only one mutation that was reported and hence these patients were not included in the analysis. Biosimulation of the malignant phenotype, also referred to as “disease induction,” requires a two-fold increase in the hallmark behaviors such as cell number, proliferation, and viability, but was not achieved in the 11/67. Finally, biosimulation requires that the disease cell comes to a steady state. However, in some cases the drug interacts with the disease network in a manner that generates positive feedback loops that prevent a steady state from being achieved (41/67)

The genomic abnormalities comprising the inputs to the model are shown in Table 1. The distribution of abnormalities is shown in Supplementary Fig. 1. Altogether 207 of 274 m*-MGMT* cancers were successfully modeled. The causes of disease induction failure are shown in Fig. 1.

**Table 1:** Genomic abnormalities identified among 274 patients with MGMT-methylated GBM

Pathway	Gene	Incidence
IDH	*IDH1*	21 (10.1%)
Homologous recombination repair deficiency (HRD)	*BRCA1*	8 (3.9%)
	*BRCA2*	62 (30.0%)
	both *BRCA1* and *BRCA2*	4 (1.9%)
	*PALB2*	21 (10.1%)
	*FANCA-D*	40 (19.3%)
	*RAD50/51*	51 (24.6%)
DNA checkpoints	*ATM*	37(17.9%)
	*ATR*	15 (7.2%)
	both *ATM* and *ATR*	4 (1.9%)
Base excision repair	*APEX1*	49 (23.7%)
	*ALKBH2/3*	48 (23.2%)
	*LIG4*	45 (21.7%)
	*XRCC3*	44 (21.3%)
	*POLB*	15 (7.2%)
Mismatch repair deficiency (MMRD)	2 genes	10 (4.8%)
	1 gene	9 (4.3%)
*TP53* abnormalities	*17p* deletion	15 (7.2%)
	*TP53* mutation	61 (29.5%)
Hedgehog pathway abnormalities	*SUFU* deletion	168 (81.2%)
	*GLI* amp	24 (11.6%)
NF-κB pathway activation	*NFKBIA* deletion	40 (19.3%)
Mitosis abnormalities	*STAG2* deletion	30 (14.5%)
Epigenetic driver abnormality	*EP300*	62 (30.0%)
	*KMT2A-D*	50 (24.2%)
	*CREBBP*	16 (7.7%)
*HOXA10*	*HOXA10* amplification	166 (80.2%)

### Efficacy predictions for single agent TMZ and lomustine

**Fig. 2:**
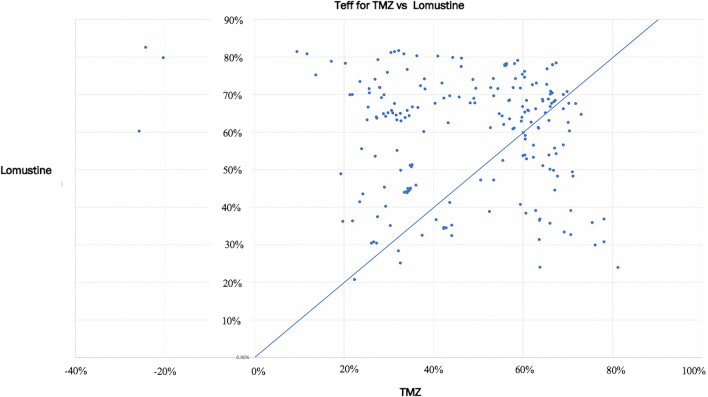
Biosimulation of drug efficacy (T_eff_) for single agents: TMZ v. lomustine. Each point represents biosimulation results for a single patient. The diagonal line represents therapeutic equivalence. Patients whose scores fall to the right of the diagonal have better predicted response from TMZ, while patients to the left of the diagonal have better predicted response from lomustine

We compared T_eff_ for TMZ alone, lomustine alone, and the combination of TMZ and lomustine. As expected for this *m-MGMT* group, almost all patients had predicted to benefit from TMZ to some degree (Range: T_eff_ − 25 to 81.78%). However, four patients had negative T_eff_ values indicating and anti-therapeutic or deleterious effect of TMZ due to the presence of mismatch repair deficiency (MMRD) which generates hypermutation without inducing lethality. Among patients with positive T_eff_ values, the magnitude of TMZ benefit varied by approximately 4.5-fold (Range: T_eff_ 17–78%). (Supplementary Fig. 1A) Interestingly, in this population of *m-MGMT* patients, lomustine showed substantial efficacy (Range: T_eff_ 20.8–82.6%). (Supplementary Fig. 1B). Comparing the two drugs head to head, 120 (57.9%) patients had higher efficacy scores from lomustine compared to TMZ as single agents. But the opposite was true for 38 (18.4%) patients who had better efficacy scores from TMZ. Roughly equivalent benefits were observed (i.e. within 5 efficacy points of each other) in 49 (23.6%) patients. (Fig. 2).

### Efficacy predictions for combination TMZ plus lomustine

The TMZ and lomustine combination generated positive T_eff_ values for all patients. This included the patients with negative T_eff_ arising from MMRD, implying that lomustine can rescue these patients from the adverse impact of TMZ in this setting. In general, the combination of TMZ and lomustine showed greater T_eff_ values than TMZ alone (Range: T_eff_ 32.44–99.99%). (Supplementary Fig. 2) A majority of patients achieved T_eff_ of approximately 78–86%, a finding supportive of the central conclusion of NOA-09.

We determined ∆T_eff_, the incremental benefit from the addition of combination therapy vs. TMZ alone varied substantially across the population (Range: T_eff_ 0.11–95.22%). (Supplementary Fig. 2A) The rectangular shape of the histogram suggests that the benefit of treatment is unpredictable, ranging from deriving nothing at all to profound benefit from the combination:

- 24 (11.6%) achieved greater than 99% efficacy, even with T_eff_ starting as low as 34% from TMZ alone.
- 9 (4.3%) would be predicted to have less than 2% increase in efficacy from the addition of lomustine.
- 38 (18.4%) would achieve less than an 11% gain from the addition of lomustine deriving little benefit from the addition of lomustine.
- 70 (33.8%) had increased T_eff_ of 11–33% from the combination, a modest benefit.
- 99 (47.8%) had greater than 33% increment in T_eff_, thus deriving substantial benefit from the combination.

We also determined ∆T_eff_ for the incremental benefit of adding combination therapy vs. lomustine alone where the combination was deleterious for some and beneficial for others (Range: T_eff_ − 50.1 to + 50.3%). (Supplementary Fig. 2B) To our surprise, the addition of TMZ detracted from T_eff_ of lomustine alone for 33 (15.9%) patients. An additional 37 (17.9%) patients had a negligible benefit from the addition of TMZ, while 64 (30.9%) had only a minimal or modest benefit, and the remainder 73 (35.4%) derived a substantial increment in phenotype response from the combination.

**Fig. 3:**
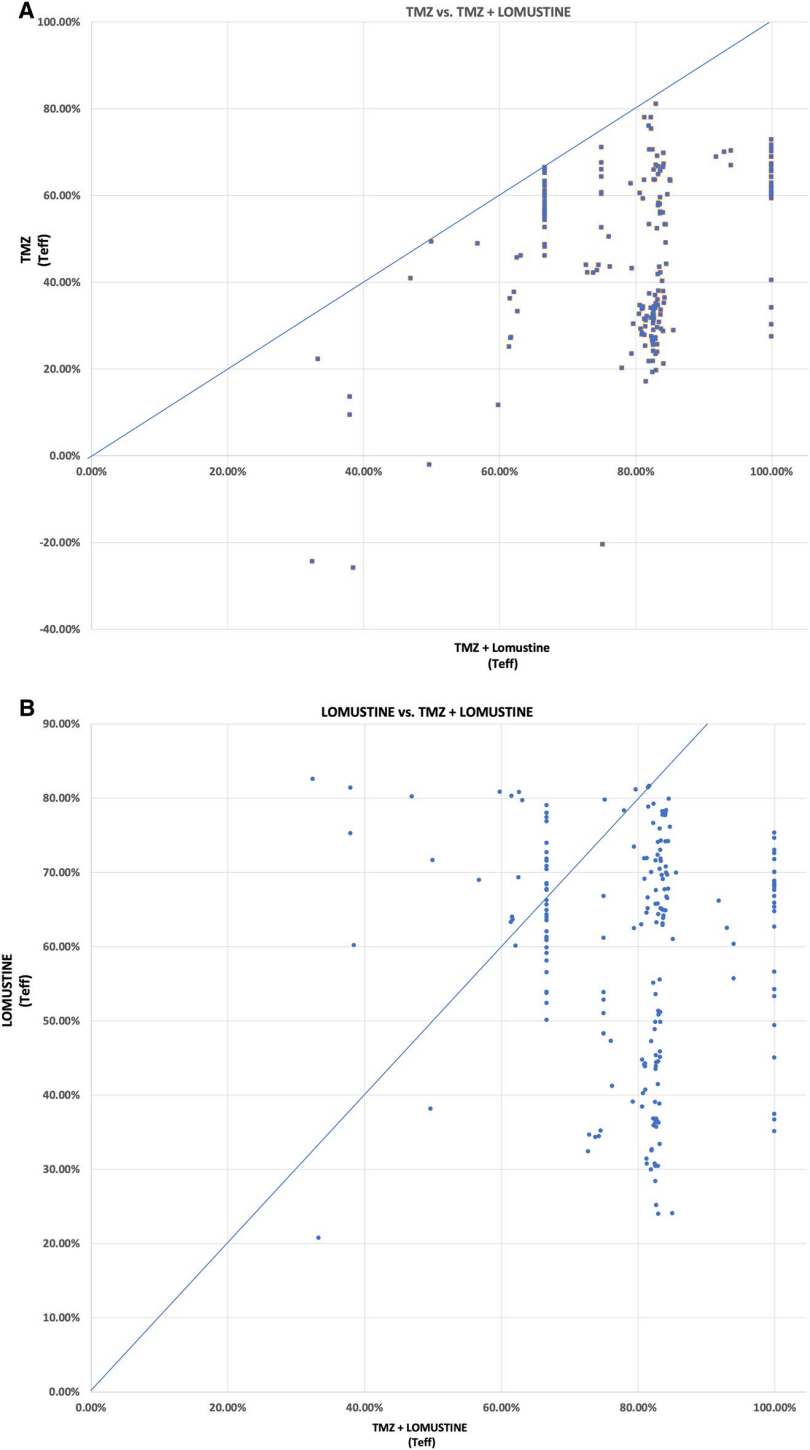
Biosimulation of drug efficacy (T_eff_) for combination v. single agent chemotherapy. Each point represents biosimulation results for a single patient. The diagonal line represents therapeutic equivalence. **A** T_eff_ scores for TMZ v. TMZ + lomustine. The horizontal distance from each point to the diagonal gives the magnitude of incremental benefit from the addition of lomustine compared to TMZ alone. **B** T_eff_ scores for lomustine v. TMZ + lomustine. The horizontal distance from each point to the diagonal gives the magnitude of incremental benefit (to the right) or decremental harm (to the left) from the addition of TMZ to lomustine compared to lomustine alone. Some patients are predicted to achieve 100% efficacy from the combination. Others who fall along the diagonal would have little or no added benefit from combination therapy compared to single agent approaches. A cluster of points reveals an incremental benefit of ~ 30% for combination therapy, while another cluster gets a smaller benefit. A few patients had TMZ values < 0 due to mismatch repair deficiency. The addition of lomustine to TMZ would be deleterious compared to lomustine alone for some patients who are above and to the left of the diagonal(3B)

**Fig. 4:**
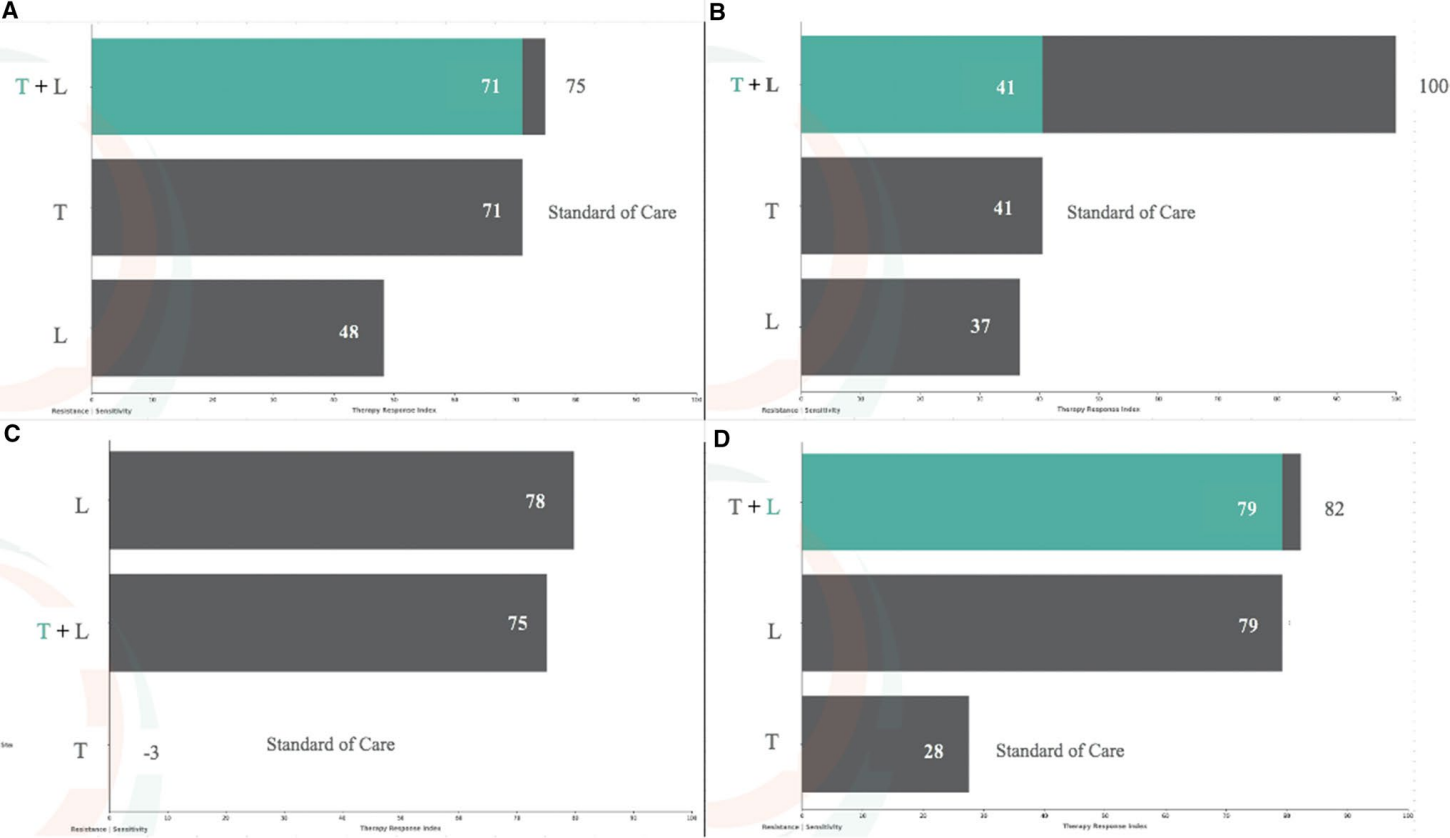
Biosimulation scenarios. Horizontal bars represent a measure of T_eff_ for T: TMZ, L: lomustine, and T + L: TMZ and lomustine. **A** TMZ is highly efficacious, superior to lomustine, and the addition of lomustine adds little value; **B** modest efficacy exists for both TMZ and lomustine, but the combination achieves > 99% efficacy; **C** lomustine is superior to TMZ; TMZ has no efficacy, and in combination detracts slightly from the benefit of lomustine alone; **D** lomustine is highly efficacious, superior to TMZ, and TMZ adds little incremental benefit

We plotted the benefit for 1) combination therapy v. TMZ alone and 2) combination therapy v. lomustine alone (Fig. 3). The diagonal line on these graphs represents equivalence for the combination (shown on the abscissa) compared to each single agent (shown on the ordinate). The horizontal distance of each point to the diagonal gives the magnitude of anticipated incremental benefit of combination therapy (Fig. 3). Patients with values close to the diagonal should not be treated with combination chemotherapy. On the other hand, a patient who falls far right of the diagonal appears ideally suited to receive combination treatment. Impressively, about one in nine patients are predicted to derive greater than 99% efficacy from combination treatment. On the other hand, for patients whose values fall in an intermediate range, clinical judgment would be needed to weigh the risk of a marginal benefit against the increased toxicity. Surprisingly, lomustine alone outperformed combination treatment in 33 (15.9%) of patients, implying that the addition of TMZ would be harmful compared to single agent lomustine and therefore contraindicated. Examples of four different response scenarios are shown for individual patients in Fig. 4 and suggest that patients should be managed with all of the treatment strategies depending on the results of biosimulation. The generalized proposition about the superiority of combination therapy for *m-MGMT* GBM though true for the population as a whole was not uniformly supported for individual patients.

## Discussion

As a rule, the benefits of chemotherapy are unevenly distributed among patients classified as having the same disease. Aside from quoting median survival and considering clinical variables (e.g. age, sex, performance status, and residual disease burden), for individual patients oncologists are at a loss to predict the magnitude of benefit of a particular chemotherapy strategy before treatment. Thus, clinical management follows the same assumptions that lead to clinical trial design and recruitment, namely that for the sake of study all *m-MGMT* patients are considered the same. But in time, patients classified as having the same disease have divergent outcomes, retrospectively making the assumption of sameness naive. By contrast, biosimulation promises to identify the *anterior probability* of treatment benefit thereby allowing physicians to tailor their clinical decision making for each patient.

In the case of TMZ and lomustine, 35–48% of *m-MGMT* patients would be predicted to get a major benefit from combination treatment compared to conventional TMZ or the alternative of single agent lomustine, perhaps justifying the inconvenience and extra toxicity. The remainder would be predicted to have either a negligible benefit or one small enough that it that might be difficult to justify considering the limited therapeutic index for some patients. With regard to the question of concurrent versus sequential therapy, some patients had relatively modest effects from each single agent, but apparent synergy when the agents were combined, thus favoring combination therapy over a sequential approach. In other circumstances, when the efficacy of both drugs was relatively high and the combination offered relatively little incremental benefit, sequential therapy would seem to be more appropriate. For some patients in this cohort, lomustine was predicted to have better efficacy as a single agent compared to TMZ, while the opposite was observed for another subgroup. One of the most surprising revelations of this study is that TMZ was predicted to cause disease progression rather regression for some and a decremental or anti-therapeutic effect when added to lomustine in 15% of patients. While these findings certainly reflect patient selection in this cohort, the results suggest caution is needed about generalizing positive conclusions from NOA-09 to individual patients.

Many other signaling pathways besides MGMT impact the efficacy and differential sensitivity to these agents suggesting that we should fight against the wish for simplicity and the preference for single biomarker rules. Biosimulation demonstrates that the complexity of signaling pathway dysregulation contributes to vast heterogeneity of drug response in this population. In the pathway analysis, a confluence of abnormalities involving NF-κB activation, hedgehog pathway activation, and MMRD generated a lack of TMZ benefit. On the other hand, HRD, base excision repair deficiency, MMRD, and epigenetic dysregulation contributed to lomustine’s predicted superiority for a subset of patients. Interestingly, MMRD, *KMT2A-D* loss, and/or *EP300* loss of function mutations or deletions produced opposite effects conferring TMZ resistance and lomustine sensitivity *in the same patient,* thus accounting for lomustine superiority over lomustine for a subgroup of patients. By contrast, HRD and *IDH1* mutations conferred sensitivity to both TMZ and lomustine. Interestingly, all the *IDH1*-mutated patients in this analysis were predicted to have a synergistic benefit from the combination treatment. However, for the majority of patients the *co-occurrence* of multiple genomic abnormalities creates an otherwise unpredictable mixture of drug responsiveness and resistance resulting in quite different treatment propositions in the clinic. As a result, the uniqueness of each GBM challenges the generalized conclusions about combination chemotherapy and creates a necessity for deeper more comprehensive molecular diagnosis that embraces the complexity of each patient’s disease and its potential divergence from generalized clinical trial conclusions.

Computational biological modeling affords not only an individualized, predictive measure of benefit, but also a quantitative one that simplifies our approach to the patient and overcomes the intrinsic complexity of deconvoluting multiple complex and potentially conflicting signaling pathway inputs. Remarkably, biosimulation embraces the uniqueness (N-of-1) and the complexity in each patient’s cancer. The current study predicts that immense variety exists in the benefit of TMZ and lomustine as single agents as well as in the magnitude of incremental benefit for adding the second drug. For the combination, biosimulation predicts the range of anticipated benefits extends from achieving more than 99% efficacy against the disease to nothing at all, thereby forging an imperative either to implement or shun the combination strategy. In effect, lomustine plus TMZ is essential medicine for some, but of no benefit at all for others, potentially much better or much worse than the average results reported in NOA-09. For a sizable population where the benefit of treatment falls between the extremes, biosimulation fosters an individualized benefit-risk definition that facilitates an informed discussion of therapeutic index, replacing bias and arbitrary opinion with personalized predictive scoring. Rather than risking under-treatment for the sake of safety or over-treatment so as not to miss the possibility for a superior outcome, biosimulation promises to empower neurooncologists to choose the most appropriate option based on the deepest understanding of each patient’s unique disease characteristics.

Our inability to biosimulate every patient with *m-MGMT* GBM represents a limitation of this approach. Approximately one-quarter of the patients in this study could not be modeled because of an insufficient number of genomic inputs, issues related to disease induction, or inability to achieve a steady state. Additionally, gaps exist in the model created by unknown consequences of genomic abnormalities which have yet to be elucidated by the research community. Implicitly, the success of any model is only as good as the completeness of the knowledge it is based on. While the CBM used in this study continues to evolve, we acknowledge the possibility that predicted outcomes of the patients who were not included in the analysis might have swayed the relative size of the subgroups identified in the study.

Another criticism of this work is that biosimulation awaits prospective clinical validation. This criticism conceals the viewpoint that aside from MGMT, the results of molecular profiling have no role to play in the management of the glioblastoma patient. Nevertheless, the proliferation of insights into the mechanisms of resistance and responsiveness, suggest this view is increasingly untenable. In fact, biosimulation is based on a scientific literature that includes many insights that are already commonplace and as such have the status of “common sense.” The association of *IDH1* mutations with TMZ responsiveness, MMRD with temozolomide failure, and the favorable impact of HRD on chemotherapy response represent examples of this. Other scientific insights are less known in clinical circles, such as the impact of HOXA10 amplification, Hedgehog pathway or NF-κB activation on TMZ failure. The integration of both commonly known and unfamiliar insights into a biosimulation model bridges the chasm between the knowledge base of cancer biology and the clinic, and vastly simplifies the labor required to assess the significance of multiple genomic findings at the point of care. We do not disagree that the model used in this study should have prospective validation in a randomized cohort who received combination therapy versus single agent chemotherapy. Rather, we propose that there is an imperative to implement knowledge buried in the cancer biology literature. The consequences for diverse patient management when that knowledge is applied create urgency to complete this validation as soon as possible. If confirmed, neurooncologists would be able to inaugurate a new era of replacing the one size-fits-all decision making and the molecular naivete of the current status quo with a computational tool that maximizes the benefits of drug resources already at hand.

In the era of comprehensive molecular diagnosis, it is likely that more effective personalized clinical management can be accomplished. The findings of this study suggest that the best therapy available is not represented by a single approach applied to all patients, but is a matter of tailoring the treatment to the molecular underpinnings in each individual’s cancer. In the patient-centric world of molecular diagnosis, the best treatment is not a winner-take-all proposition, but a personalized approach derived from deep insights about the biology of each individual’s disease. The Bayesian approach to clinical decisions based on biosimulation creates the possibility of deriving actionable insight from comprehensive molecular diagnosis to fulfill the mission of giving the correct treatment to every patient. Lastly, molecular diagnosis often points to novel treatment possibilities for subgroups of patients. These include connecting cancers with HRD or *STAG2* deficiency with PARP inhibitors, epigenetic dysregulation with histone deacetylase or EZH2 inhibitors, and DNA checkpoint abnormalities with ATM or ATR inhibitors, to name a few. The refinement of patient selection for the next generation of clinical trials based on deep molecular interrogation and comprehensive signaling pathway modeling promises greater success than has been part of the neuro-oncology journey so far. As such, biosimulation provides a practical solution for immediate patient care, but also the opportunity to evolve the armamentarium beyond conventional cytotoxic therapy and illuminate the next steps in the creation of individualized precision therapy for glioblastoma.

## Supplementary Information

Below is the link to the electronic supplementary material.

Supplementary file1 (DOCX 1058 kb)

## Abbreviations

*ATM*: ATM serine/threonine kinase
*ATR*: Ataxia telangiectasia and Rad3-related protein
BER: Base excision repair
CBM: Computational biological modeling
*EZH2*: Enhancer of zeste homolog-2
GBM: Glioblastoma
*HOXA10*: Homeobox protein A-10
*IDH1*: Isocitrate dehydrogenase-1
HRD: Homologous recombination repair deficiency
HRR: Homologous recombination repair
*KMT2*: Lysine methyltransferase-2
*MGMT*: O^6^-methylguanine-DNA methyl-transferase
MMR: Mismatch repair
MMRD: Mismatch repair deficiency
NHEJ: Non-homologous end joining
PARP: Poly ADP-ribose phosphorylase
STAG2: Cohesin subunit SA-2
TCGA: The Cancer Genome Atlas
TMZ: Temozolomide
