# Supplementary material for: Combination chemotherapy versus temozolomide for patients with methylated MGMT (m-MGMT) glioblastoma: results of computational biological modeling to predict the magnitude of treatment benefit
Source: J Neurooncol. 2021 Jun 8;153(3):393–402. doi: 10.1007/s11060-021-03780-0 (PMC8280043; doi:10.1007/s11060-021-03780-0)
Supplement: Supplementary file 1 — Supplementary file1 (DOCX 1058 kb) [file 11060_2021_3780_MOESM1_ESM.docx]

***SUPPLEMENTARY SECTIONS Contents***

1. Biosimulation
2. Figures

**SUPPLEMENTARY SECTION 1 Biosimulation**

A novel Cellworks Omics Biology Model contains information acquired from a variety of data sources, including studies on cell receptors, signaling pathways, transcription factors, activation factors, and enzyme kinetics. The model was developed and validated using PubMed to generate patient-specific protein network maps.^[^^[[1]](#endnote-1),^^[[2]](#endnote-2),^^[[3]](#endnote-3),^^[[4]](#endnote-4)]^ To ensure accuracy of computational simulation models, published data was aggregated through manual scientific review. Simulation experiments were employed to develop a predictive tumor model and validated in cell line experiments.

The simulation model includes representations of key signaling pathways underlying growth factor signaling, cell cycle regulation, tumor metabolism, oxidative stress, epigenetics, protein homeostasis, DNA damage repair, and apoptosis. The current version of the model includes more than 3,765 genes, including 286 kinases, 379 transcription factors, 115 pathways, and 29,181 functional interactions associated with signaling pathways associated with cancer.

A health cell model was developed from protein-protein interactions that are simulated mathematically using Michaelis-Menten equations until the system reached homeostasis. Subsequently, a virtual disease model based on somatic gene mutations and gene copy number variations (CNVs) from individual patients is modeled to generate phenotype behaviors associated with malignancy. The computational model derives a composite score representing cell number from individual phenotype scores representing hallmark behaviors of cancer, including proliferation, survival, apoptotic blockade, genomic instability, invasion, angiogenesis, and immune evasion.

The quantitative impact of individual next generation sequencing results on patient-specific protein networks is computationally modeled to determine the consequences of a drug or drug combination on the signaling networkGenomic sequencing, gene expression, and/or cytogenetic data for 207 m-MGMT GBM patients were collected from cbioportal database (https://www.cbioportal.org/). Key assumptions are made indicating the aberrations in each patient’s disease network: gain of function or amplification of tumor promoter genes, and loss of function or deletion of tumor suppressor genes drives cancer. ^[^^[[5]](#endnote-5)]^ Gene variants with therapeutic implications were assessed using public domain literature to determine each mutation’s functionality, represented as either a loss or gain of function. However, genes with mutations of unknown significance were parsed through a suite of variant calling algorithms to determine if the mutation is deleterious. For deleterious mutations, tumor promoter genes were assumed to have gain of function while tumor suppressor genes were assumed to have loss of function at the protein activity level. Unless otherwise annotated, frameshift mutations were assumed to cause a loss in gene function.

For CNV interpretation, amplifications were represented as an increase of gene expression while deletions were represented as knockdown of gene expression. Additionally, amplifications of tumor suppressor genes were considered to have lower contribution to the disease when compared to amplification of tumor promoter genes. A deletion of tumor suppressor genes was considered to have a higher dominance in the disease network when compared to deletion of tumor promoter genes.

Protein network maps were created based on each patient unique mutational profile, incorporating intersecting protein networks when multiple genomic abnormalities were detected.

**SUPPLEMENTARY SECTION 2 FIGURES**

**Supplementary Figure 1:** Incidence of chromosomal and mutation abnormalities


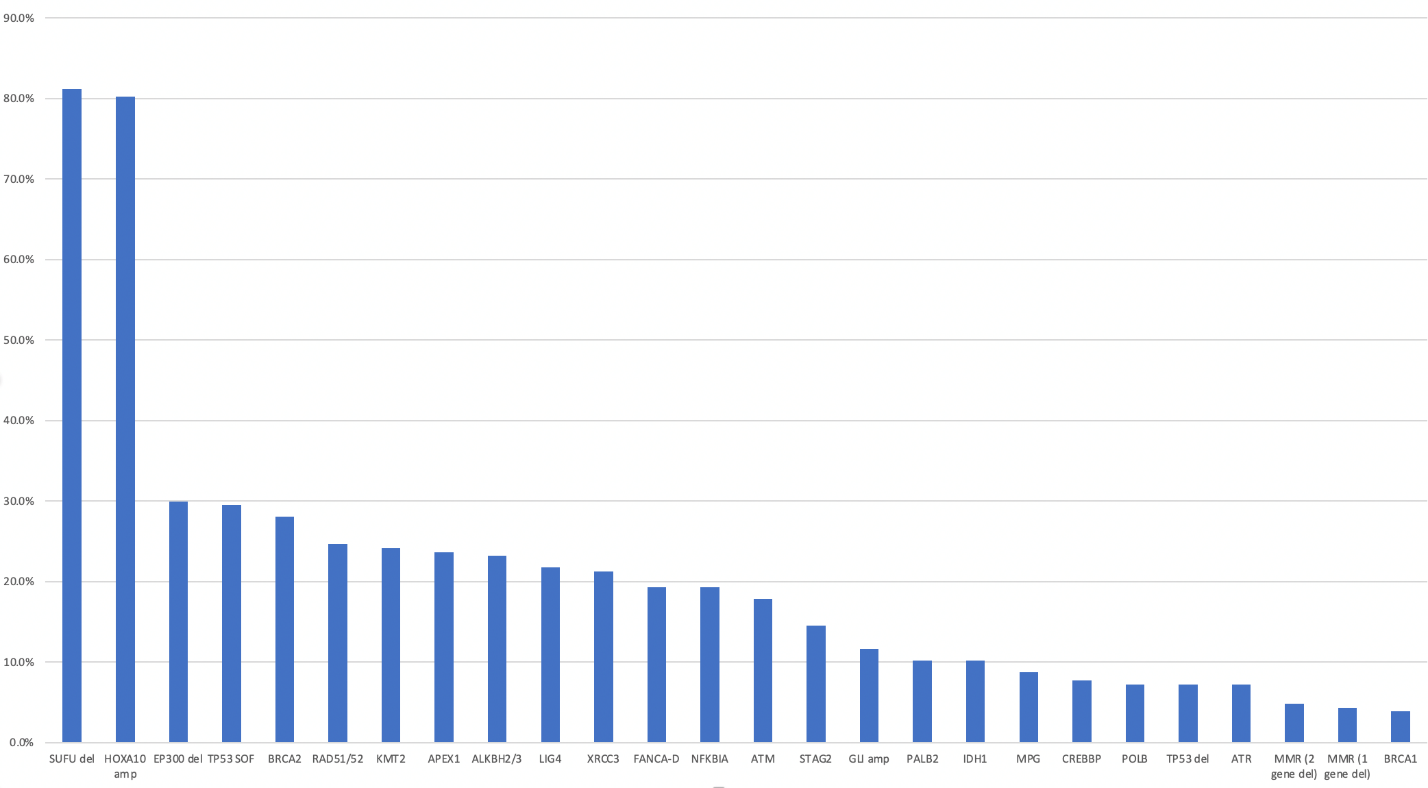


**Supplementary Figure** **2:** Distribution of treatment efficacy (T_eff_) among GBM patients with *m-MGMT* for (A) temozolomide and (B) lomustine and (C) TMZ and lomustine.

A.


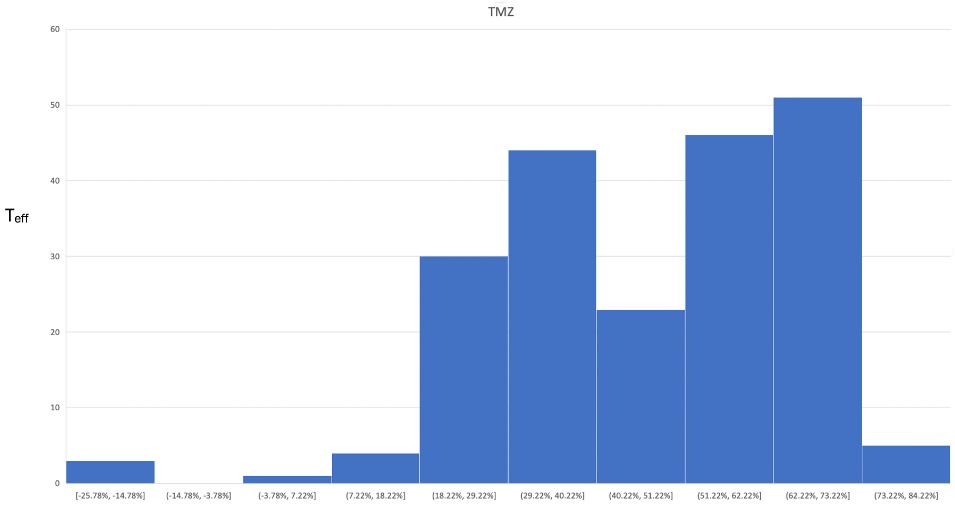


T_eff_

B.


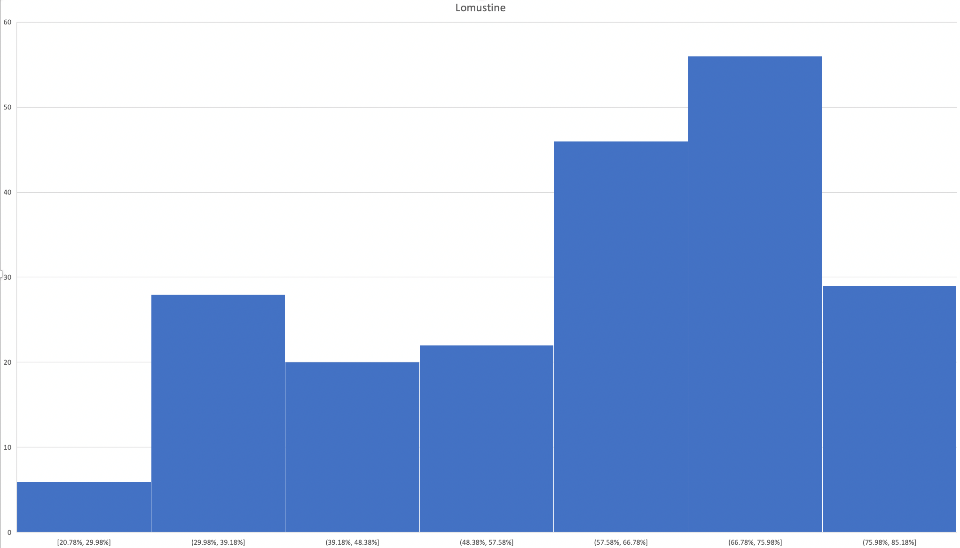


T_eff_

C.


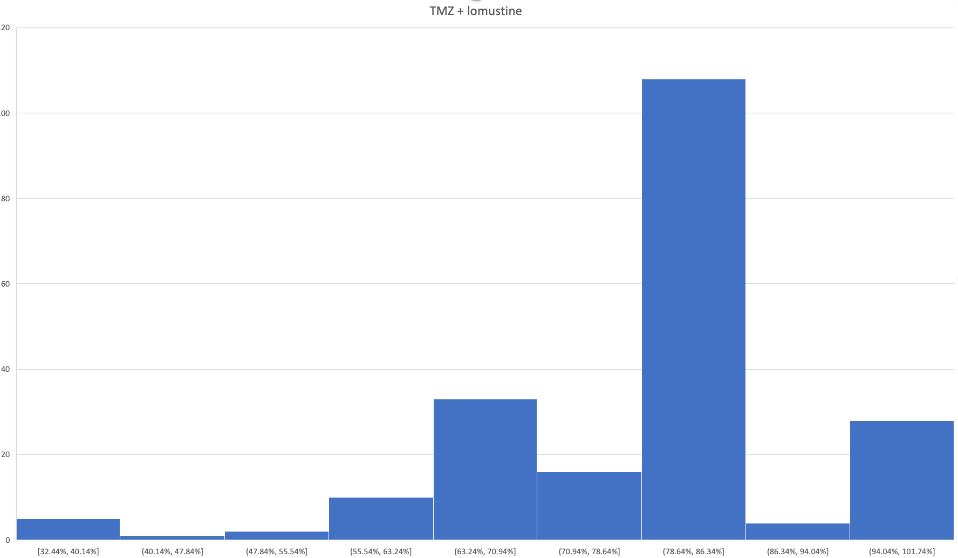


T_eff_

**Supplementary Figure 3:** (A) Distribution of the incremental value of combination TMZ plus lomustine v. TMZ alone; (B) Distribution of the incremental value of combination TMZ plus lomustine v. lomustine alone.

**A.**


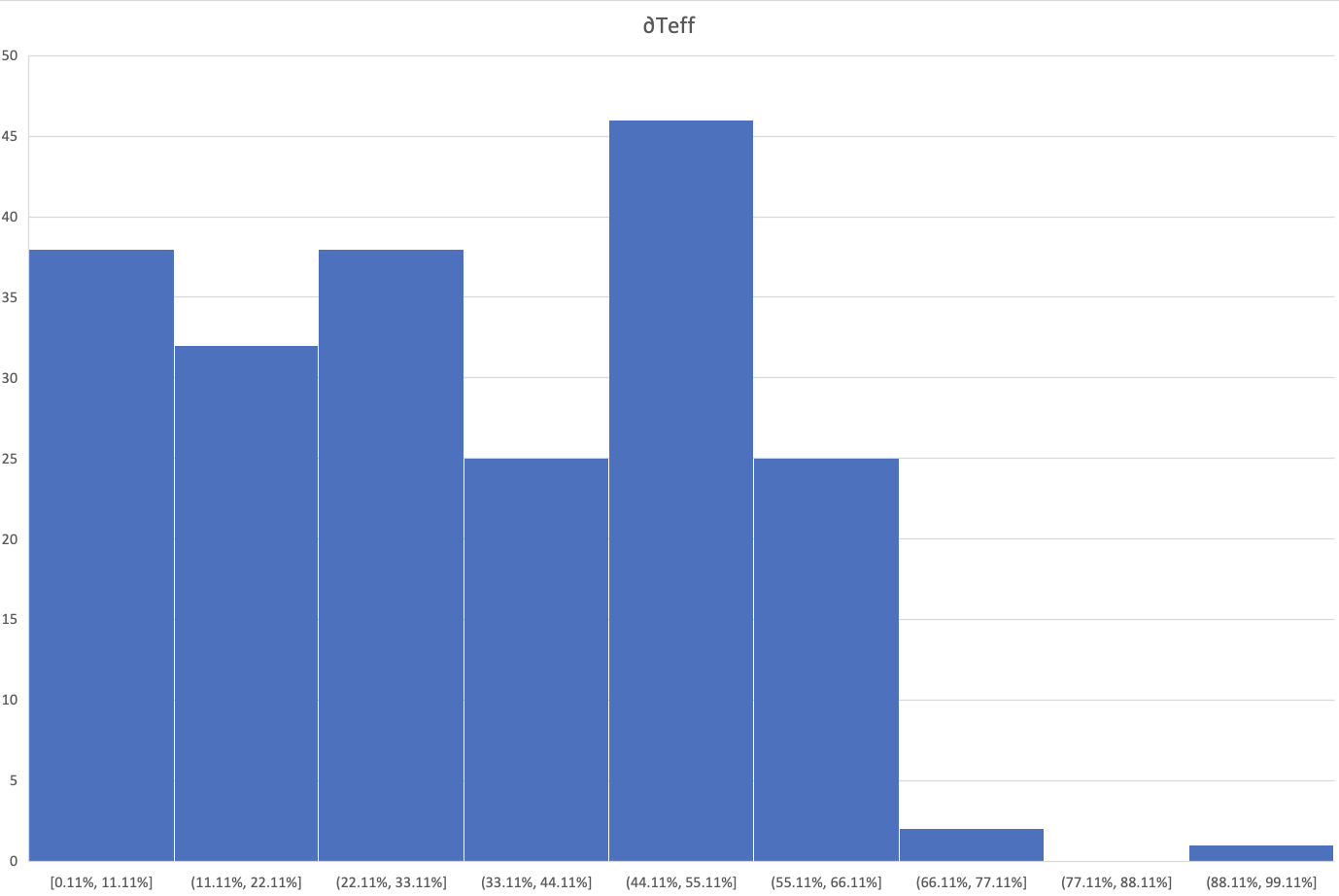


% change

**B.**


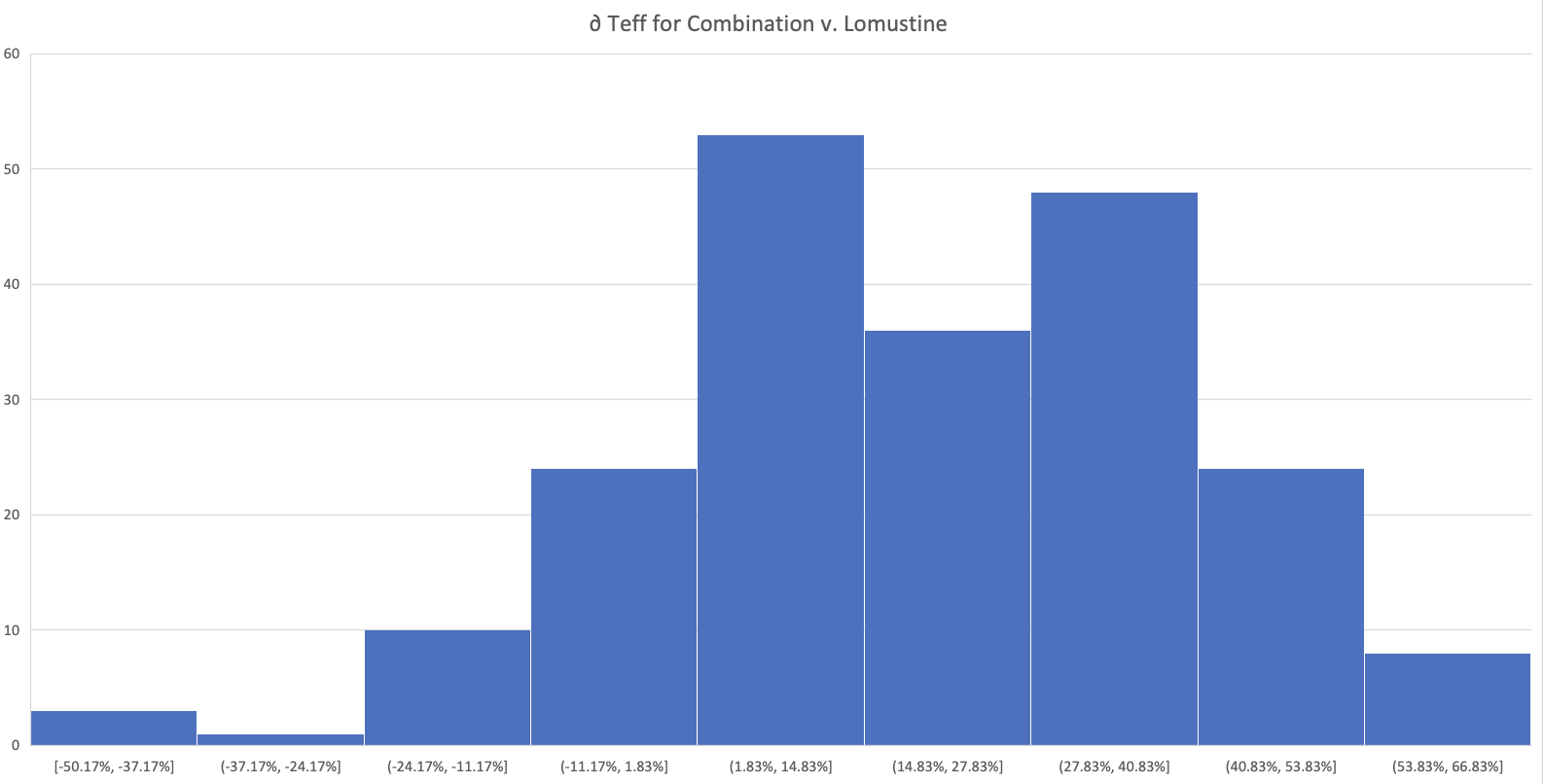


% change

% change

1. Drusbosky LM, Medina C, Martuscello R, Hawkins KE, Chang M, Lamba JK, et al. Computational drug treatment simulations on projections of dysregulated protein networks derived from the myelodysplastic mutanome match clinical response in patients. Leuk. Res., 2017. 52:1-7 [↑](#endnote-ref-1)
2. Drusbosky LM, Cogle CR. Computational modeling and treatment identification in the myelodysplastic syndromes. Curr. Hematol. Malig. Rep. 2017. p. 14 [↑](#endnote-ref-2)
3. Doudican NA, Kumar A, Singh NK, Nair PR, Lala DA, Basu K, et al. Personalization of cancer treatment using predictive simulation. J Transl Med. 2015;13:43. doi: 10.1186/s12967-015-0399-y. [↑](#endnote-ref-3)
4. Pingle SC, Z. Sultana Z, S. Pastorino S, P. Jiang, R. Mukthavaram, Y. Chao, et al. In silico modeling predicts drug sensitivity of patient-derived cancer cells. J. Transl. Med., 12 (2014), p. 128 [↑](#endnote-ref-4)
5. Kavianpour M, Ahmadzadeh A, Shahrabi S, Saki N. Significance of oncogenes and tumor suppressor genes in AML prognosis. Tumour Biol. 2016. 37(8):10041-52. doi: 10.1007/s13277-016-5067-1. [↑](#endnote-ref-5)
